# O2.8. TRAJECTORIES OF NEUROCOGNITIVE FUNCTIONING OVER TIME IN YOUTH AT CLINICAL HIGH RISK WHO DO AND DO NOT TRANSITION TO PSYCHOSIS

**DOI:** 10.1093/schbul/sby015.198

**Published:** 2018-04-01

**Authors:** Kristen Woodberry, William S Stone, Daniel I Shapiro, Cole M Chokran, Jean Addington, Carrie Bearden, Kristin Cadenhead, Tyrone D Cannon, Barbara A Cornblatt, Thomas H McGlashan, Daniel H Mathalon, Diana O Perkins, Ming T Tsuang, Elaine F Walker, Scott W Woods, Larry J Seidman

**Affiliations:** 1 Beth Israel Deaconess Medical Center, Harvard Medical School; 2 Beth Israel Deaconess Medical Center; 3 University of Calgary; 4 University of California, Los Angeles; 5 University of California, San Diego; 6 Yale University; 7 Zucker Hillside Hospital; 8 University of California, San Francisco; 9 University of North Carolina, Chapel Hill; 10 University of California, San Diego, Institute of Genomic Medicine, University of California, La Jolla; 11 Emory University

## Abstract

**Background:**

In spite of evidence for the premorbid and prodromal onset of cognitive deficits in schizophrenia and related psychotic disorders, there is some limited evidence to suggest that deficits may progress with psychosis onset. Cognitive remediation in youth at risk for psychosis is being touted as an opportunity not only to remediate deficits but to potentially prevent this progression. Yet trajectories of cognitive functioning over time remain poorly understood in youth at risk, including the degree to which age at assessment or illness onset, sociodemographic factors, or symptom progression influence these trajectories.

**Methods:**

The North American Prodrome Longitudinal Study (NAPLS) -2 collected data on an extensive battery of neuropsychological (NP) tests at baseline, one year, two years, and post-conversion in a sample of clinical high risk (CHR) youth and healthy comparison (HC) subjects ages 12–35 (N= 960, 92% of the full sample) followed clinically for up to 2 years. NP data were available for 694 at CHR and 265 HC. Linear mixed effects analyses were used to test the effects of group, age, gender, age of onset, maternal education, and clinical outcome on cognitive trajectories.

**Results:**

Those who transitioned to a psychotic disorder over the course of follow-up performed significantly below those who did not and well below healthy comparisons. Tasks reliant on attention, visual and auditory working memory, visuospatial and verbal memory, and processing speed best differentiated those who transitioned from those who did not at one year (Cohen’s d from -0.33 to -0.54). Discrepancies from normal functioning on these tests were generally large (Cohen’s d from -0.67 to -1.02) consistent with findings for first episode samples. Although clinical outcome was not associated with a significantly different trajectory over time on any cognitive domain, these are likely due to high rates of conversion in this sample within the first year. Predictors of different trajectories will be presented.

**Discussion:**

These data from one of the largest CHR studies to date suggest that much of the neuropsychological dysfunction in major psychotic disorders is present early in the course of illness and prior to its full expression. However, trajectories are highly heterogeneous. More frequent assessment prior to and during the onset of illness are needed to fully understand the cognitive correlates of psychosis onset and the implications for early intervention.

